# Functional repertoire, molecular pathways and diseases associated with 3D domain swapping in the human proteome

**DOI:** 10.1186/2043-9113-2-8

**Published:** 2012-04-03

**Authors:** Khader Shameer, Ramanathan Sowdhamini

**Affiliations:** 1 National Centre for Biological Sciences (TIFR), GKVK Campus, Bangalore 560065, India; 2 Division of Cardiovascular Diseases, Mayo Clinic, Rochester, MN 55905, USA

**Keywords:** Protein aggregation, Human disease, Deposition disease, Human proteome, Data integration, Biological data mining

## Abstract

**Background:**

3D domain swapping is a novel structural phenomenon observed in diverse set of protein structures in oligomeric conformations. A distinct structural feature, where structural segments in a protein dimer or higher oligomer were shared between two or more chains of a protein structure, characterizes 3D domain swapping. 3D domain swapping was observed as a key mediator of numerous functional mechanisms and play pathogenic role in various diseases including conformational diseases like amyloidosis, Alzheimer's disease, Parkinson's disease and prion diseases. We report the first study with a focus on identifying functional classes, pathways and diseases mediated by 3D domain swapping in the human proteome.

**Methods:**

We used a panel of four enrichment tools with two different ontologies and two annotations database to derive biological and clinical relevant information associated with 3D domain swapping. Protein domain enrichment analysis followed by Gene Ontology (GO) term enrichment analysis revealed the functional repertoire of proteins involved in swapping. Pathway analysis using KEGG annotations revealed diverse pathway associations of human proteins involved in 3D domain swapping. Disease Ontology was used to find statistically significant associations with proteins in swapped conformation and various disease categories (*P-value *< 0.05).

**Results:**

We report meta-analysis results of a literature-curated dataset of human gene products involved in 3D domain swapping and discuss new insights about the functional repertoire, pathway associations and disease implications of proteins involved in 3D domain swapping.

**Conclusions:**

Our integrated bioinformatics pipeline comprising of four different enrichment tools, two ontologies and two annotations revealed new insights into the functional and disease correlations with 3D domain swapping. GO term enrichment were used to infer terms associated with three different GO categories. Protein domain enrichment was used to identify conserved domains enriched in swapped proteins. Pathway enrichment analysis using KEGG annotations revealed that proteins with swapped conformations are present in all six classes of KEGG BRITE hierarchy and significantly enriched KEGG pathways were observed in five classes. Five major classes of disease were found to be associated with 3D domain swapping using functional disease ontology based enrichment analysis. Five classes of human diseases: cancer, diseases of the respiratory or pulmonary system, degenerative diseases of the central nervous system, vascular disease and encephalitis were found to be significant. In conclusion, our study shows that bioinformatics based analytical approaches using curated data can enhance the understanding of functional and disease implications of 3D domain swapping.

## Background

Computationally efficient classification, annotation and prediction algorithms are rapidly improving our understanding of protein sequence-structure-function relationships. Analysis of such relationships often helps in our understanding of novel sequence or structural features in the regulation of a particular function including molecular pathways and various disease mechanisms. Cells attain its functional integrity with the help of molecular mechanisms including protein-protein interactions [1-7]. Protein folding and subsequent oligomerization of protein chains help such interactions in cellular environment. Protein-protein interactions play a key role in mediating higher order oligomerization. Protein-protein interactions are diverse in nature and they can be broadly classified, as transient interactions where the interactions are weak and obligatory interactions that are permanent in nature. Based on sequence homology, two proteins with high degree of similarity could interact and form a homodimer, where as two distantly related proteins could form a heterodimer [8,9]. 3D domain swapping is a unique protein structural mechanism observed in homodimers or higher order oligomers with a specific type of interaction, where a segment of two protein chains are mutually swapped. 3D domain swapping was also observed in protein structures in heteroligomer conformations. 3D domain swapping was associated with several proteins that were involved in diverse functional events and disease pathways. Previous studies on 3D domain swapping using structural properties indicated that 3D domain swapping share similar structural features of oligomeric protein complexes and primarily associated with deposition diseases [10-13]. Prior studies on 3D domain swapping were focused on small set of proteins largely due to the unavailability of a curated database of proteins involved in 3D domain swapping. In this study, we present results from analysis of proteins in the human genome and curated in 3DSwap knowledgebase using multiple biological enrichment methods. 3DSwap is the first database that catalogued proteins involved in 3D domain swapping. The database was developed using a *literature-based protein structural curation *strategy that utilized manual curation and a structural bioinformatics pipeline to gather data pertaining to 3D domain swapping. We used complete set of human proteins from 3DSwap database and examined statistically significant domains, biological process, cellular component, molecular function, biological pathways and diseases using enrichment methods. From a bioinformatics perspective, this manuscript is a case study that leverage application of robust bioinformatics methods to gain new functional and therapeutic insights from a protein structural mechanism.

### 3D domain swapping: Pathophysiological basis of deposition diseases

**Figure 1:**
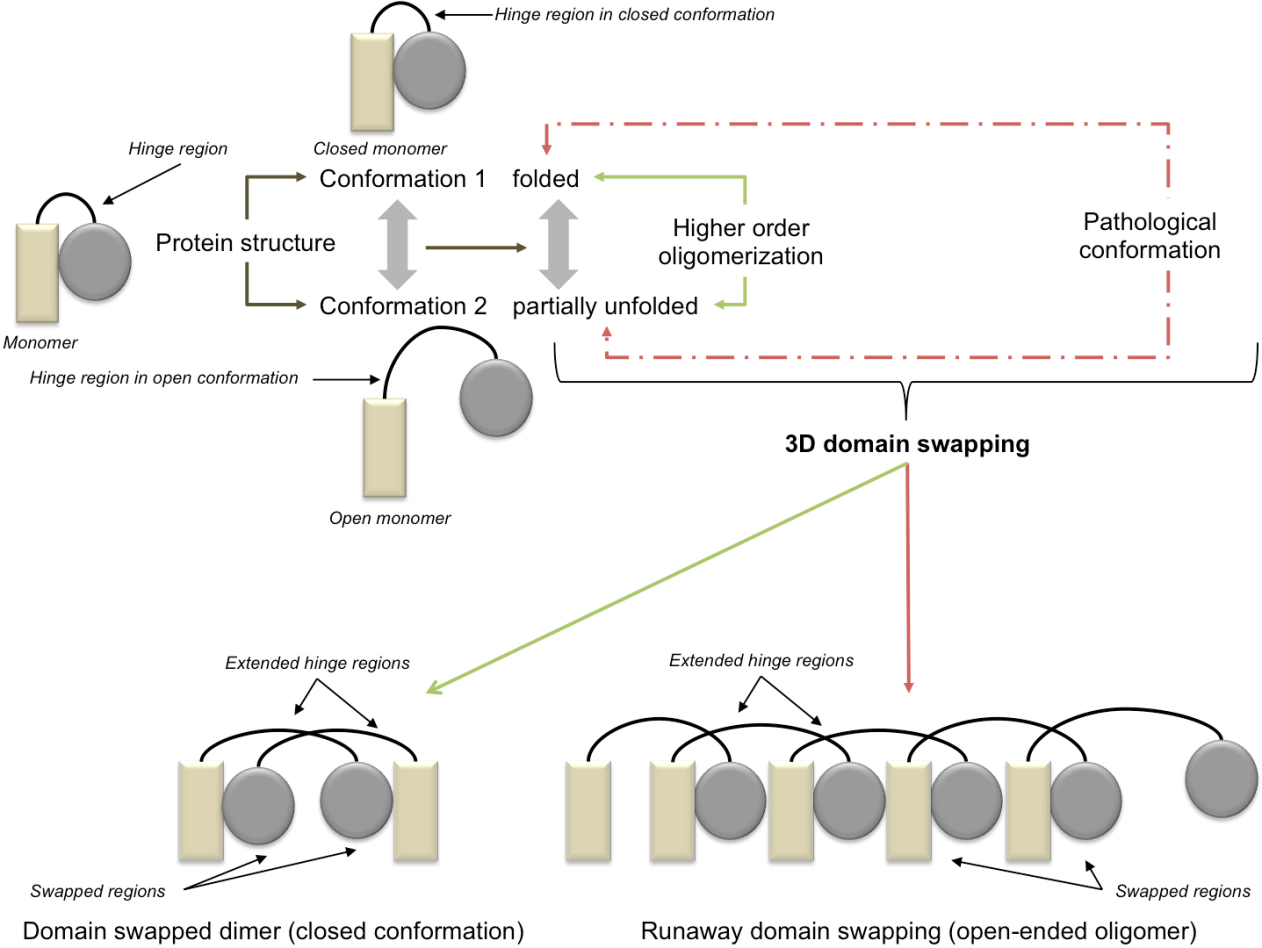
**Schematic representation of 3D domain swapping**.

3D domain swapping is a unique protein structural phenomenon with implications in function, form and disease (Figure 1). Only two scenarios (domain swapped dimer and open-ended oligomeric swapping) of 3D domain swapping are provided in the figure. Other scenarios like double domain swapping, cyclic swapping and entirely swapped structures were observed in proteins with swapped oligomeric architecture. Protein structures involved in 3D domain swapping is characterized by hinge regions and swapped regions. 3D domain swapping is associated with mutual swapping of a structural segment between two or more chains in a protein oligomer. This mechanism was observed in a diverse group of proteins that mediate different structural, functional and physiological mechanisms. 3D domain swapping was primarily defined as a mechanism for functional or structural oligomeric assembly, recently defined as the molecular mechanism behind protein aggregation and thus implicated as a pathogenic basis of diseases like deposition diseases or conformational diseases [14], amyloidosis [15], serpinopathies [16] and proteinopathies [16]. Proteins involved in such diseases have higher aggregation propensities and involved in the formation of highly specific aggregates of a single protein. From a structural perspective, some of these aggregates were generated by 3D domain swapping mechanism [12-14,17-33]. From a clinical perspective, such diverse disease manifestations mediated by this single structural mechanism are of great interest. It still remains elusive whether 3D domain swapping is exclusively associated with such conformational diseases or they may also play a crucial role in mediating complex diseases.

### Dataset of human proteins involved in domain swapping

Irrespective of numerous biochemical and computational studies focused on the molecular basis of 3D domain swapping [11,34-52], a detailed account of functional repertoire, including protein domains, Gene Ontology (GO) terms, biological pathways and disease associated with proteins in swapped conformation, were not reported. The mechanism of 3D domain swapping was reported in different evolutionary lineages and structures in swapped conformation were identified in multiple organisms with a large proportion characterized from eukaryotes. Hitherto, proteome-wide analysis of this unique structural mechanism was impossible due to the non-availability of proteome level curated dataset. Recently, we integrated in-depth literature curation and structural bioinformatics analytics to curate proteins involved in 3D domain swapping from Protein Data Bank (PDB) and reported a knowledgebase of proteins involved in 3D domain swapping [53]. 3DSwap offers a compendium of 293 protein structures with delineated hinge regions, swapped regions and offers an ideal resource to study functional and structural implications of domain swapping.

### Inference from biological and biomedical ontologies using enrichment analysis

Enrichment analysis plays an important role in knowledge-based bioinformatics approaches [54,55]. In this study, enrichment analysis was performed using annotations derived from Pfam domains [56], GO [57-59], KEGG pathways [60] and Disease Ontology (DO) [61,62]. Enrichment analysis in bioinformatics is a collective term referring to a group of statistical bioinformatics algorithms developed to understand the global trends of a subset of genes or gene products compared to a background population (for example, all genes in the human genome and whole proteins encoded in the entire human genome or all genes tested in a given experiment or genes included in gene expression platforms etc.). Huang et al. [54] suggested a nomenclature to classify enrichment tools in bioinformatics as singular enrichment analysis (SEA), gene set enrichment analysis (GSEA) [63] and modular enrichment analysis (MEA) [55]. Fundamental differences between these three classes of algorithms arise in the manner by which the enrichment *P-value *was calculated. In SEA-based approach, annotation terms of subset of genes were assessed one at a time against a list of background genes. An enrichment *p*-value was calculated by comparing the observed frequency of an annotation term with the frequency expected by chance and individual terms beyond the *p*-value cut-off (*P*-value ≤ 0.05). BiNGO [64], FunctAssociate [65], Onto-express [66,67] are examples of SEA-based enrichment analysis tools. GSEA approaches are similar, but consider all genes during the enrichment analysis, instead of a pre-defined threshold based genes, as in SEA approach. For example, Gene Ontology terms are connected by relationships and MEA based programs like Ontologizer [68] and topGO [69] employ the relationships that exist between the annotations. These programs were reported to attain better sensitivity and specificity due to the consideration of GO term relationships. GSEA is an enrichment-based computational method to determine whether an a priori defined set of genes show statistically significant differences, when compared between two biological states [63]. For example, a set of human genes differentially regulated in a gene expression of analysis for a particular type of cancer can be considered as a prior gene list, and the background can be defined one or more datasets compiled in Molecular Signatures Database (MSigDB) [70]. A variety of tools are currently available for the functional enrichment analysis, a recent review cited 69 tools for such analysis and the list of tools are rapidly growing. Majority of these tools employ statistical methods using Fisher's test [71,72], hypergeometric function [64], binomial test [72] or *χ^2 ^*tests [73] or combination of such methods as implemented in tools like GFINDER [74] and Onto-Express [66,67] for significant association of the GO terms and the gene list with respect to the background distribution. Concept of gene set enrichment analysis was incorporated in to various programs that use biological or functional annotations of genes and gene products to perform biological enrichment calculations using ontologies and annotations. Gene Ontology enrichment and pathway enrichment analysis employ similar conceptual and statistical methods to understand functional and molecular roles of subset of genes or proteins were found to be very efficient in summarizing functional diversity or similarity trends. Such approaches are routinely employed in gene expression studies, high-throughput screening experiments and genome-wide association studies (GWAS) [75,76].

Gene ontology enrichment and pathway enrichment analysis, using ontologies or annotations derived from a subset of genes characterized from an experimental or computational study, generally applied to infer new biological insights, which was otherwise impossible with candidate gene-centric approaches. Due to the generic nature of statistical methods used in enrichment analysis, current set of enrichment algorithms and related statistical methods can be used to infer enrichment from annotation databases. Enrichment calculations are currently available for various types of annotations. Annotations of protein domains (Pfam [56], SMART[77]), pathways (KEGG [60], GenMAPP[78]) and human gene-disease associations using Online Mendelian Inheritance in Man (OMIM) [79] are currently used for enrichment analysis. Similar to GO, any ontology (for example: disease ontology (DO) [62]) maintained by Open Biological and Biomedical Ontologies (OBO) [80] foundry or its mapping or derivatives (for example: disease-ontology (DOLite) [61]) can be effectively used for enrichment analysis.

### Enrichment tools, ontologies, annotation databases and statistical methods

This study utilized four *tools*, two *ontologies *and two *annotation databases *for inferring functional and disease insights from list of human proteins involved in 3D domain swapping. Protein domain enrichment was performed using DAVID 6.7. Protein domain annotations were derived from Pfam database, a database of evolutionarily conserved protein domain coordinates. Ontologizer 2.0, a GO term enrichment tool with command-line interface and improved statistical method for deriving GO terms enriched in a given list of proteins was used in this study. SubPathwayMiner, an R package that internally handles KEGG annotations for pathway enrichment analysis were used to derive statistically significant pathways associated with the dataset. Enriched disease ontology terms were identified using Functional Disease Ontology server that consults Disease Ontology and it's derivative disease-ontology lite for identifying significant diseases. *H_0 _*= List of curated proteins with swapped conformations are not associated with any class of protein domains, gene ontology terms, KEGG pathways or disease ontology terms. We tested our null hypothesis individually using four different tools and associated annotations or ontologies. *P-value *from enrichment analyses were obtained using default statistical settings of different tools employed in this study. Protein domain enrichment *P-values *were derived from DAVID using a modified Fisher Exact P-value, called EASE score [81]. GO term enrichment analysis *P-values *were derived using Ontologizer 2.0 and corrected using Bonferroni method [68]. KEGG pathway enrichment using SubPathwayMiner, it provides False Discovery Rate (FDR) corrected *P-values*. Disease enrichment analysis was performed using Functional Disease Ontology server and it uses a Fisher's exact test for deriving *P-values*.

## Methods

### Curated dataset of human proteins involved in 3D domain swapping

**Figure 2:**
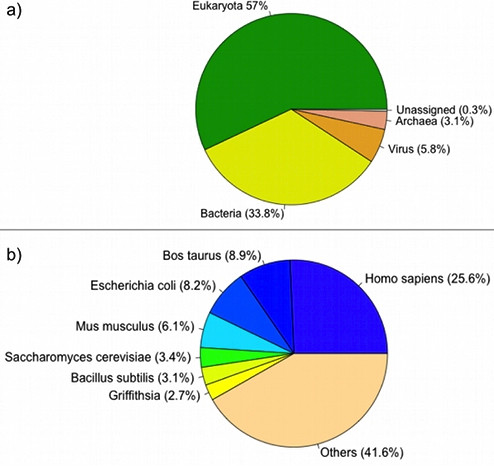
**Taxonomic (a) and species (b) level distribution of proteins in swapped conformation from 3DSwap knowledgebase**.

**Figure 3:**
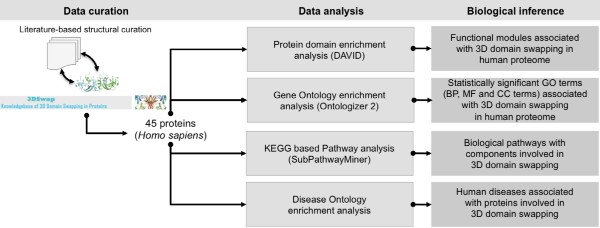
**Bioinformatics pipeline employed to derive functional, pathway and diseases associations of proteins involved in 3D domain swapping**.

Classification of proteins in 3DSwap knowledgebase based on SOURCE record from PDB and subsequent mapping using SIFTS annotations revealed that 75 structures out of 293 structures reported in 3DSwap were from *Homo sapiens*. A cursory look at 3DSwap database for the taxonomic spread would indicate that the largest fraction was from humans (25.6%) (Figure 2). We used literature-curated structures from 3DSwap database with delineated 'hinge' and 'swapped' regions for the analysis in (see Additional file 1: Supplementary Table 1) for list of proteins used in this study). 75 PDB identifiers were mapped to UNIPROT and KEGG database identifiers using Protein ID cross-reference (PICR) service and custom Perl scripts [82]. Out of the 75 curated protein structures with 3D domain conformation retrieved from 3DSwap knowledgebase, 45 proteins were unique (See Table 1). Human proteins from our curated dataset had several redundant structures. To avoid potential functional bias, only unique human proteins (45/75 structures) were used in this analysis. Graphical summary of the bioinformatics pipeline employed in this study is depicted in Figure 3.

**Table 1:** Enriched Pfam domains associated with proteins involved in 3D domain swapping

Pfam identifier	Pfam Description	*P-value*
PF07714	Protein tyrosine kinase	3.0E-6

PF00031	Cystatin domain	1.1E-5

PF01463	Leucine rich repeat C-terminal domain	1.9E-3

PF00625	Guanylate kinase	3.3E-4

PF07679	Immunoglobulin I-set domain	6.6E-3

### Enrichment analysis of human proteins involved in 3D domain swapping

Protein domain enrichment analysis was performed using DAVID [81]. KEGG pathway analysis was performed using SubPathwayMiner [83] and Disease Ontology analysis was performed using Functional Disease Ontology server [61,62].

### Protein domain enrichment analysis

To perform protein domain enrichment analysis, domains were identified in proteins involved in 3D domain swapping and a list of protein domains was obtained. This list of protein domains was compared against a reference dataset of protein domains associated with complete human proteome. Protein domain enrichment analysis was performed to understand statistically significant, conserved, functional modules associated with proteins involved in 3D domain swapping. Dataset of 45 Uniprot identifiers were used for protein domain enrichment analysis using Pfam annotations. DAVID version 6.7 with default settings was used for the analysis.

### Gene ontology enrichment analysis

GO term enrichment analysis in this study was performed using Ontologizer 2.0, a multifunctional tool for GO term enrichment analysis. Ontologizer was selected due to the improved statistical approximation methods incorporated in it. A brief description of the method is provided here. Generic GO enrichment tools calculate the enrichment of a GO term with respect to the list of genes in the dataset and the background population using the probability of drawing the same or higher number of genes annotated to a given term. This basic concept was implemented using statistical test involving the upper tail of the hypergeometric distribution or one-tailed Fisher's exact test. Such methods do not consider relationships between the annotation terms. GO is defined as a directed acyclic graph (DAG), with various levels of relationships between the terms. Due to DAG architecture of GO, a gene or gene product annotated with a term *x *is also annotated to all parent terms of *x*, and this often leads to false enrichment calculations. Such relationships (for example: *is a*, *part of*, *has part*, *regulates*) were taken into account in Ontologizer 2.0 using parent-child inheritance concepts [84]. Detailed description about the statistical method implemented in the Ontologizer 2.0 can be found elsewhere [68,84]. Dataset consisting of 45 Uniprot identifiers were used for species (*Homo sapiens*) specific GO enrichment analysis and pathway analysis. GO enrichment analysis was performed using the following parameters using Ontologizer 2.0: Gene Ontology annotations were derived from human-specific annotation data (gene_association.goa_human) [58], multiple testing correction was set to "Bonferroni correction" method, enrichment calculation was set to Parent-child-Intersection, re-sampling step was set to 1000. Gene Ontology was defined using 33,738 terms and 59,508 relations recorded in the gene_ontology.obo file (downloaded on February 2011) were used for the analysis. Background population for statistical tests was defined using 18,257 proteins encoded in the human genome with Gene Ontology annotations.

### KEGG based pathway enrichment analysis of proteins in human proteome with swapped conformation

Pathway enrichment analysis using KEGG pathway annotations were performed to understand the role of proteins in 3D domain swapping conformation in various biological pathways. UNIPROT Identifiers were mapped to Entrez gene identifiers using custom Perl scripts and used as the input in R package SubPathwayMiner [83] for pathway enrichment analysis. Pathways associated with these proteins were obtained from KEGG pathway database and compared to a reference database of full list of proteins and its corresponding pathways annotated in KEGG databases.

### Disease enrichment analysis of proteins in swapped conformation using disease ontology

The disease ontology tem enrichment analysis was performed using Functional Disease Ontology server [62]. List of 45 human genes mapped to UNIPROT Identifiers were mapped to Entrez gene identifiers using custom Perl scripts. List of Entrez identifiers were used as input for Disease Ontology enrichment to understand the role of the human proteins with swapped conformation in various biological pathways. Out of 45 genes in the list, 35 were found to be associated with at least one disease. Briefly, the disease association of each gene in the human genome was annotated using the Disease Ontology and peer-reviewed evidence from Gene Related Information into Function (GeneRIF) [61,62,85]. A condensed version of the Disease Ontology, Disease Ontology Lite [61], was used for the statistical analysis. Similar to Gene Ontology analysis, the significance of each disease association was evaluated using Fisher's exact test.

## Results

3D domain swapping is a structural mechanism employed by a variety of protein structures to form oligomeric assemblies. These oligomers were often associated with aggregation diseases or proteinopathies in humans. Parkinson's diseases and Alzheimer's diseases are two major neurodegenerative diseases due to phenotypic impact of 3D domain swapping. Hitherto, no comprehensive study has been reported to analyze the impact of all proteins involved in 3D domain swapping from a whole proteome-wide or genome-wide perspective due to unavailability of a well-defined, curated dataset. We performed the initial investigation of proteins involved in 3D domain swapping in the level of protein domains, Gene Ontology, KEGG pathways and Disease Ontology. Our approach helped to understand enriched protein domains, Gene Ontology terms, biological pathways and Disease Ontology terms mediated by these proteins and their role in mediating various human diseases.

Statistically significant protein domains associated with swapped proteins in the human proteome is provided (Table 1), GO terms (Tables 2, 3, 4), KEGG pathways (Table 5) and DO terms (Table 6), associated with swapped proteins encoded in the human proteome, are provided. Critical aspects of statistically significant evolutionarily conserved domains, GO terms, KEGG pathways and DO terms associated with human proteins in swapped conformation are summarized in the 'Discussion' section.

**Table 2:** Statistically significant *Biological Process *terms from GO term enrichment analysis

GO ID	GO term	*P-value*
GO:0048518	Positive regulation of biological process	0.002

GO:0016032	Viral reproduction	0.002

GO:0048519	Negative regulation of biological process	0.005

GO:0009987	Cellular process	0.006

GO:0040007	Growth	0.008

GO:0018126	Protein amino acid hydroxylation	0.008

GO:0032501	Multicellular organismal process	0.009

GO:0035110	Leg morphogenesis	0.01

GO:0007154	Cell communication	0.01

GO:0016271	Tissue death	0.011

GO:0051704	Multi-organism process	0.014

GO:0090046	Regulation of transcription regulator activity	0.014

GO:0050896	Response to stimulus	0.015

GO:0044403	Symbiosis, encompassing mutualism through parasitism	0.015

GO:0001775	Cell activation	0.016

GO:0065007	Biological regulation	0.017

GO:0023052	Signaling	0.019

GO:0032502	Developmental process	0.021

GO:0034465	Response to carbon monoxide	0.021

GO:0014071	Response to cycloalkane	0.023

GO:0006793	Phosphorus metabolic process	0.023

GO:0051098	Regulation of binding	0.026

GO:0000003	Reproduction	0.032

GO:0045342	MHC class II biosynthetic process	0.033

GO:0001816	Cytokine production	0.037

GO:0008356	Asymmetric cell division	0.037

GO:0046417	Chorismate metabolic process	0.038

GO:0030431	Sleep	0.038

GO:0048610	Reproductive cellular process	0.039

GO:0007610	Behaviour	0.043

**Table 3:** Statistically significant *Cellular Component *terms from GO term enrichment analysis

GO ID	GO term	*P-value*
GO:0005802	Trans-Golgi network	0.002

GO:0071944	Cell periphery	0.004

GO:0005737	Cytoplasm	0.009

GO:0045121	Membrane raft	0.024

GO:0048786	Presynaptic active zone	0.05

**Table 4:** Statistically significant *Molecular Function *terms from GO term enrichment analysis

GO ID	GO term	*P-value*
GO:0060089	Molecular transducer activity	0.008

GO:0003682	Chromatin binding	0.008

GO:0042802	Identical protein binding	0.011

GO:0019838	Growth factor binding	0.011

GO:0046983	Protein dimerization activity	0.011

GO:0004713	Protein tyrosine kinase activity	0.013

GO:0019144	ADP-sugar diphosphatase activity	0.02

GO:0004883	Glucocorticoid receptor activity	0.023

GO:0030545	Receptor regulator activity	0.035

GO:0050998	Nitric-oxide synthase binding	0.047

GO:0001871	Pattern binding	0.048

GO:0070851	Growth factor receptor binding	0.049

**Table 5:** KEGG pathways associated with proteins involved in 3D domain swapping in the dataset.

KEGG Pathway ID	Pathway Name	*P-value*	KEGG BRITE class
hsa05200	Pathways in cancer	0.000	Human Diseases; Cancers

hsa04722	Neurotrophin signaling pathway	0.000	Organismal Systems; Nervous System

hsa05144	Malaria	0.000	Human Diseases; Infectious Diseases

hsa04630	Jak-STAT signaling pathway	0.000	Environmental Information Processing; Signal Transduction

hsa05120	Epithelial cell signaling in Helicobacter pylori infection	0.000	Human Diseases; Infectious Diseases

hsa05211	Renal cell carcinoma	0.000	Human Diseases; Cancers

hsa04510	Focal adhesion	0.001	Cellular Processes; Cell Communication

hsa04660	T cell receptor signaling pathway	0.001	Organismal Systems; Immune System

hsa05310	Asthma	0.002	Human Diseases; Immune System Diseases

hsa04060	Cytokine-cytokine receptor interaction	0.002	Environmental Information Processing; Signaling Molecules and Interaction

hsa05020	Prion diseases	0.002	Human Diseases; Neurodegenerative Diseases

hsa05330	Allograft rejection	0.003	Human Diseases; Immune System Diseases

hsa00620	Pyruvate metabolism	0.003	Metabolism; Carbohydrate Metabolism

hsa04672	Intestinal immune network for IgA production	0.005	Organismal Systems; Immune System

hsa05320	Autoimmune thyroid disease	0.006	Human Diseases; Immune System Diseases

hsa05110	Vibrio cholerae infection	0.006	Human Diseases; Infectious Diseases

hsa05221	Acute myeloid leukemia	0.006	Human Diseases; Cancers

hsa04144	Endocytosis	0.008	Cellular Processes; Transport and Catabolism

hsa05218	Melanoma	0.009	Human Diseases; Cancers

hsa05100	Bacterial invasion of epithelial cells	0.009	Human Diseases; Infectious Diseases

hsa05220	Chronic myeloid leukemia	0.010	Human Diseases; Cancers

hsa04520	Adherens junction	0.010	Cellular Processes; Cell Communication

hsa00400	Phenylalanine, tyrosine and tryptophan biosynthesis	0.010	Metabolism; Amino Acid Metabolism

hsa04664	Fc epsilon RI signaling pathway	0.012	Organismal Systems; Immune System

hsa05222	Small cell lung cancer	0.013	Human Diseases; Cancers

hsa04012	ErbB signaling pathway	0.014	Environmental Information Processing; Signal Transduction

hsa04210	Apoptosis	0.014	Cellular Processes; Cell Growth and Death

hsa04540	Gap junction	0.015	Cellular Processes; Cell Communication

hsa04010	MAPK signaling pathway	0.018	Environmental Information Processing; Signal Transduction

hsa05146	Amoebiasis	0.020	Human Diseases; Infectious Diseases

hsa04360	Axon guidance	0.029	Organismal Systems; Development

hsa04530	Tight junction	0.031	Cellular Processes; Cell Communication

Statistically significant associations are highlighted in bold

**Table 6:** Disease ontology terms associated with proteins involved in 3D domain swapping.

DO Term	Genes	*P-value*
**Asthma**	***IL10, TJP1, BCL2L1, IL5 ***	**0.001**

**Amyotrophic lateral sclerosis**	***MET, DCTN1, CST3 ***	**0.001**

**Bronchial hyperreactivity**	***IL10, IL5 ***	**0.001**

**Pulmonary alveolar proteinosis**	***IL10, CST3 ***	**0.001**

**Dental plaque**	***IL10, TJP1, BCL2L1 ***	**0.002**

**Prostate cancer**	***IL10, NCOA2, GLO1, SERPINC1, CST3 ***	**0.002**

**Fatty liver**	***MET, IL10 ***	**0.003**

**Atherosclerosis**	***NOD1, IL10, EPHX2, CST3 ***	**0.003**

**Rabies**	***RNASE1, BCL2L1, SERPINC1 ***	**0.004**

**Parkinson disease**	***IL10, EPHX2, BCL2L1 ***	**0.004**

**Thyroid cancer**	***IL10, TJP1 ***	**0.023**

**Neoplasm metastasis**	***IL10, RNASE1, CST3 ***	**0.024**

**Hypertension**	***IL10, EPHX2, CST3 ***	**0.028**

Breast cancer	*IL10, NCOA2, CSTA, CST3 *	0.05

Lung cancer	*IL10, CSTA, CSTB *	0.057

Adenovirus infection	*PTK2, BCL2L1 *	0.072

Abortion	*NOD1, IL10 *	0.085

Autistic disorder	*MET, GLO1 *	0.096

Kidney disease	*PTK2, SERPINC1 *	0.101

Kidney failure	*IL10, CST3 *	0.128

Enteritis	*NOD1, IL10 *	0.142

Autoimmune disease	*IL10, BCL2L1 *	0.148

Systemic scleroderma	*MET, IL10 *	0.173

Ulcerative colitis	*NOD1, IL5 *	0.18

Multiple sclerosis	*GLO1, BCL2L1 *	0.184

Infection	*NOD1, IL10 *	0.266

Dermatitis	*CSTA, IL5 *	0.294

Cancer	*MET, PTK2, EPHX2, BCL2L1 *	0.329

Lupus erythematosus	*IL10, PTK2 *	0.378

Melanoma	*IL10, TJP1 *	0.41

Alzheimer's disease	*IL10, CST3 *	0.713

Embryoma	*IL10, CST3 *	0.99

Rheumatoid arthritis	*BCL2L1, CST3 *	0.99

Colon cancer	*NOD1, TJP1 *	0.99

Leukemia	*IL10, NCOA2 *	0.99

Diabetes mellitus	*TJP1, CST3 *	0.99

Statistically significant associations are highlighted in bold

Proteins involved in 3D-domain swapping represents a large collection of proteins with a variety of functional and regulatory roles in the cell. Due to limitation in crystallizing structures in the swapped conformation, currently available repertoire of proteins in the swapped conformation may represent only a small fraction of proteins that may perform its molecular role *via *3D domain swapping. Machine learning algorithms and computational approaches may help to predict more proteins with features of 3D domain swapping [11,52]. Here we discuss primary insights obtained from the initial investigation of proteins involved in 3D domain swapping. Present results from the human proteome indicates an important paradigm that future drug design studies, focusing on various disease categories or pathways associated with 3D domain swapping, should consider the structural implications of this important structural mechanism and associated mechanisms like macromolecular crowding and protein aggregation.

### Functional repertoire of proteins involved in 3D domain swapping

Protein domain enrichment analysis reveals that five protein domain families were enriched in the dataset (See Table 1). These include protein tyrosine kinase domain, a member of kinase domain family involved in signal transduction [86], cystatin domain, a member of cysteine protease inhibitor family [87], leucine-rich repeat C-terminal domain, an unique motif that mediates protein-protein interaction [88], Guanylate kinase, a key mediator of catalytic reaction that converts adenosine triphosphate (ATP) to adenosine diphosphate (ADP) and adenosine monophosphate (AMP) [89] and Immunoglobulin I-set domain found in several cell adhesion molecules [90]. We noted that significantly enriched conserved protein domains associated with 3D domain swapping plays pivotal role in various signaling pathways, thus it also points the role of domain swapping in multiple signal transduction events.

### Statistically significant GO terms associated with swapped proteins

**Figure 4:**
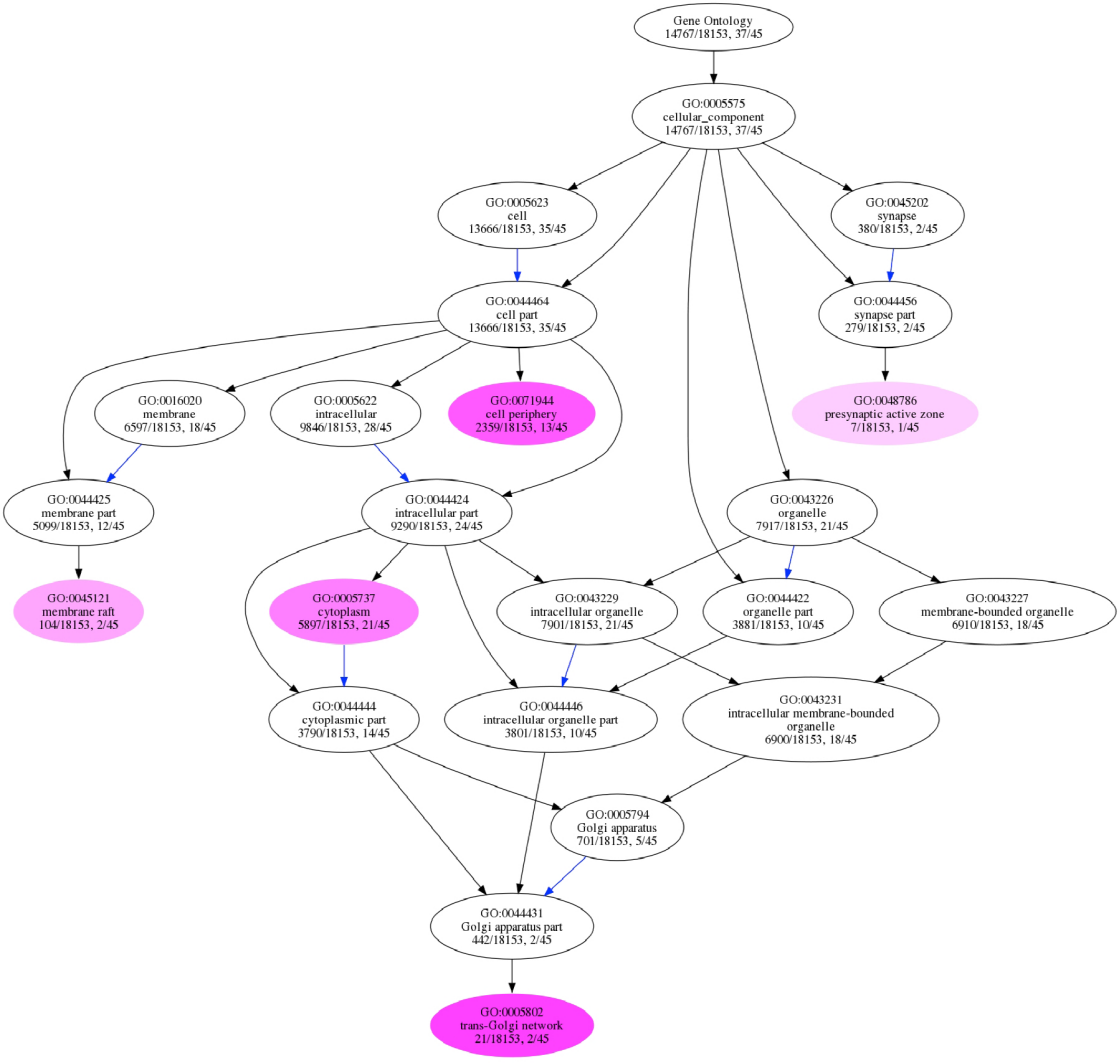
**Gene Ontology enrichment analysis (Cellular Component) using unique human proteins from the dataset**. Colored nodes indicate enriched terms associated with proteins involved in 3D domain swapping.

GO term enrichment analysis revealed that multiple terms in three different GO categories were associated with swapped proteins encoded in the human proteome. This includes 31 GO terms in biological process category (Table 2), five GO terms in cellular component category (Table 3) and 12 terms in molecular function category (Table 4). DAG structure with highlighted GO terms in biological process (Additional file 1: Figure S1), cellular compartment (Figure 4) and molecular function (Additional file 1: Figure S2) categories are provided. Biological process contains several non-specific and specific GO terms that point towards functional understanding of the proteins involved in 3D domain swapping. Top "Biological Process" terms include viral reproduction and protein amino acid hydroxylation. Two cellular transport related terms under "Cellular Component" category (membrane raft and trans-Golgi network), along with cytoplasm and cell periphery, were also found to be associated with human proteins involved in 3D domain swapping. Enriched molecular function terms indicate that human proteins involved 3D domain swapping is involved in multiple signaling and binding activities including chromatin binding, protein kinase activity and protein dimerization activity. This also indicates specific role of proteins involved in swapping and its association with mechanisms like oligomerization, macromolecular crowding and aggregation which are considered to be cellular mechanisms implicated by 3D domain swapping. GO term enrichment analysis provided a cursory view of biological processes, cellular components and molecular functions associated with 3D domain swapping.

### Implications of 3D domain swapping in in biochemical pathways

**Figure 5:**
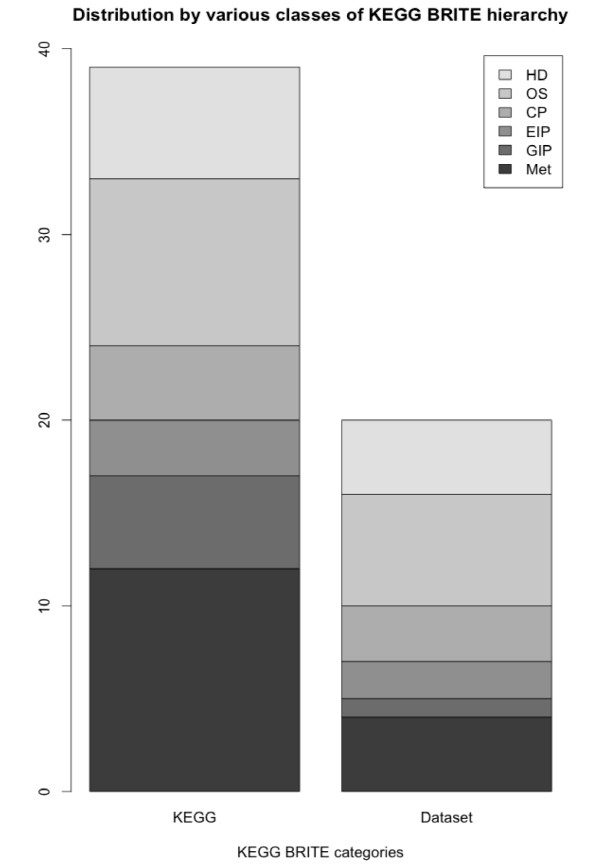
**Comparison of KEGG BRITE hierarchies in KEGG database and proteins from the human dataset mapped to KEGG BRITE hierarchy**. HD = Human Diseases, OS = Organismal Systems; CP = Cellular Processes; EIP = Environmental Information Processing; GIP = Genetic Information Processing and Met = Metabolism.

Results from pathway enrichment analysis using BioConductor based SubPathwayMiner package indicates that proteins in swapped conformation participate in multiple biological pathways. Results from pathway enrichment analysis using KEGG annotations are provided in Table 5. KEGG database classifies the pathways using a top-level functional hierarchy classification using KEGG-BRITE hierarchy. According to this hierarchy, human pathways were classified into six categories (Metabolism, Genetic Information Processing, Cellular Processes, Organismal Systems and Human diseases). Current analysis reveals that proteins with 3Dswap conformations are present in all six classes, but significantly enriched KEGG pathways were observed in all classes except the Genetic Information Processing. Proteins involved in 3D domain swapping are observed in multiple subcategories of KEGG pathway hierarchy (see Figure 5). KEGG pathway analysis indicated that proteins in the swapped conformation are statistically significant in four subclasses of human disease class viz. Cancers, Immune System Diseases, Infectious Diseases and Neurodegenerative Diseases. Proteins are also involved in other subclasses of diseases like Cardiovascular Diseases of KEGG BRITE hierarchy (See Table 5).

### Disease implications of proteins involved in 3D domain swapping

**Figure 6:**
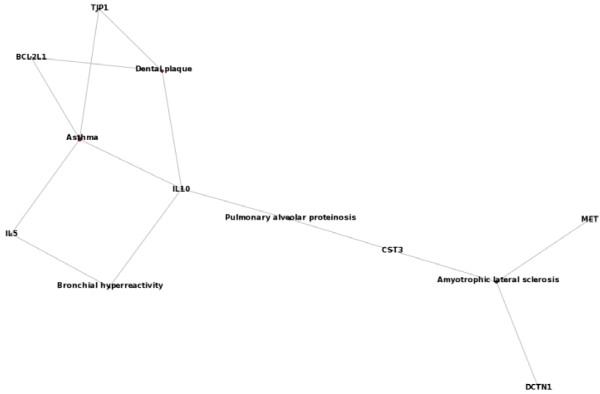
***Disease Ontology term - gene *network derived from Disease Ontology enrichment analysis using human proteins involved in 3D domain swapping**.

Since KEGG pathways represent biochemical pathways and disease pathways in a single framework, a further detailed analysis of human proteins in swapped conformation was performed using a dedicated ontology that defines human diseases. Functional disease ontology annotation tool that uses Disease Ontology-derived "Disease Ontology-lite" and GeneRIFs were used in this analysis due to the brevity of the terms and availability of significant gene-disease association data. Enrichment analysis using disease ontology provided a detailed overview of the statistically significant association between gene-products in the swapped conformation with various disease categories. Using the current subset of data, five major classes of diseases were observed in the disease Ontology-based enrichment analysis as follows: cancer (prostate cancer, thyroid cancer, breast cancer and neoplasm metastasis), diseases of the respiratory or pulmonary system (asthma, bronchial hyperreactivity, pulmonary alveolar proteinosis), degenerative diseases of the central nervous system (Amyotrophic lateral sclerosis, Parkinson's Disease), vascular disease (atherosclerosis, hypertension) and encephalitis (rabies). Neurodegenerative diseases are well-known to have strong association with 3D domain swapping, but insights into other diseases indicates that there could be more proteins with disease association and 3D domain swapping, beyond the currently well-known group of conformational diseases. Detailed table with Disease Ontology term (disease), genes associated with each disease and *P-value *for the association is provided in Table 6. Five of the significantly enriched diseases in the dataset and the genes associated with the diseases are provided as a network (Figure 6). Network is defined using genes as nodes and disease shared between the genes are considered as common edge between two genes. Disease ontology is useful to map disease relationships across human genes and diseases. To expand this disease association to clinically relevant information, we curated the disease ontology terms associated with 3D domain swapping to derive the associated International Classification of Diseases - 9 (ICD-9) codes. Diseases under the following ICD-9 codes 001-139 (infectious and parasitic diseases), 140-239: (neoplasms), 320-359 (diseases of the nervous system), 390-459: diseases of the circulatory system, 460-519 (diseases of the respiratory system). This further helped to understand major classes of clinically relevant disease phenotypes mediated by a unique molecular mechanism.

## Discussion

Domain swapping is a key pathophysiological mechanism mediating conformational disease. A detailed account of functional repertoire, molecular pathways and spectrum of diseases affected by this mechanism remains elusive. We used enrichment calculations to understand the aspects using a curated dataset of proteins involved in 3D domain swapping. Our analysis was performed using a dataset of 45 unique human proteins derived from 3DSwap knowledgebase [53]. This dataset will be growing in the future as structural characterization of human proteins involved in domain swapping is rapidly increasing. Numerous structures are being identified and more proteins with swapped conformation may found to be associated with domain swapping. Performing analysis using the approaches we employed in the future may help to identify additional protein domains, Gene Ontology terms, molecular pathways and human diseases.

Due to oligomeric features of swapping, earlier studies have indicated that 3D domain swapping plays a crucial role in conformational diseases or deposition diseases and proteinopathies. There was limited insight on structure-function relationship of proteins involved in domain swapping due to unavailability of a large dataset to objectively analyze functional or disease implications implicated by 3D domain swapping. Proteins encoded in the human genome and reported to be involved in 3D domain swapping were analyzed in detail to understand the role of gene products in various classes of diseases, beyond conformation diseases or proteinopathies. Mapping and enrichment analysis of human proteins involved in 3D domain swapping to KEGG pathways in 'disease' class and Disease Ontology indicates that these proteins play a significant role in various other diseases categories along with well-known neurodegenerative or conformational diseases.

Availability of genome-scale sequence data and annotations were considered as the ideal resource for gaining new insights from a plethora of biological data. Structural mechanisms can gain new insights about the functional aspects by mapping and database-wide enrichment analysis using annotations. In a similar way, functional mechanism may also gain new insight by using knowledge-based approaches employed in this study. In summary, the present study reports the application of knowledge-based approaches to understand new functional insights about a structural mechanism. Starting from an initial dataset of protein structures, the present study shows the importance and impact of the data integration and data mining to derive biologically relevant interpretations of global trends of a structural mechanism from sequence, functional and disease perspective. Further new insights are obtained from a translational perspective by focusing on proteins involved in 3D domain swapping in the human genome. 3D domain swapping is a unique phenomenon and may affect availability of active sites and binding sites required to impart the biological function depending on the swapped conformation. Perhaps, future drug design studies should consider these important aspects while developing therapeutics for various disease categories where 3D domain swapping is observed.

### Clinical relevance of 3D domain swapping

In the current era of personal genomes and network medicine, clinical and therapeutic approaches are utilizing integrated approaches for the understanding of disease states and pathophysiological mechanisms. Complex disease states are often triggered by perturbations in multiple pathways by multiple genes [91-94]. Protein structures and structural mechanisms play an important role in the phenotypic impact of various diseases and signaling pathways [95-101]. Protein structural information is routinely utilized to identify drug targets that will help in development of effective drugs [102-104]. New approaches will be required to target proteins or biochemical pathways with proteins in the swapped conformation. Our study illustrates the application of biological and biomedical enrichment tools, ontologies and annotations to understand functional role and disease implications of an important structural mechanism from the global perspective of human proteome.

Insights obtained from our disease ontology analysis indicates that 3D domain swapping is not just confined to neurodegenerative diseases, proteins in swapped conformation play a significant role in several other classes of diseases like cancer, vascular disease, pulmonary disease etc. Enrichment results discussed in this paper will be useful in such studies in the future from biochemical, functional, structural and therapeutic perspective. Our analysis also indicates that further genome-specific analysis of proteins involved in 3D domain swapping, using comparative genome analysis framework, may also add further understanding of functional, structural and pathophysiological manifestations of 3D domain swapping.

## Conclusion

3D domain swapping is an important structural mechanism associated with a diverse set of proteins involved in multitude of biological processes and molecular functions and diseases including proteinopathies. This phenomenon is often studied from the perspective of protein structure and its impact on biological pathways, correlations with biological functions and association with classes of diseases other conformational diseases were largely unknown. We performed a knowledge-based analysis of human proteins involved in 3D domain swapping to find the key functions, pathways and diseases associated with 3D domain swapping. Our study was limited to 45 unique proteins involved in 3D domain swapping. 3D domain swapping is a functionally relevant phenomenon due to its primary role in protein oligomerization; proteins with swapped oligomeric states are being identified on a regular basis using crystallography experiments. Effective algorithms that can predict swapping from structural and sequence information may also help to identify more proteins in swapped confirmation. As more proteins are being characterized in swapped conformation, performing such knowledge-based analysis using new proteins, improved annotations and enhanced ontologies may reveal additional functional classes, pathways and disease. In summary, we showed results from an initial investigation to understand conserved protein domains, functional repertoire, pathways and diseases mediated by 3D domain swapping in human proteome.

## Competing interests

The authors declare that they have no competing interests.

## Authors' contributions

KS curated the data, performed the analysis and compiled the first draft of the manuscript. RS conceived the project, designed the curation strategy, discussed the approaches and provided critical comments to the manuscript. All authors read and approved the final manuscript.

## Supplementary Material

Additional file 1 Supplementary Table 1 Click here for file

Additional file 2 Figure S2 Click here for file

**Additional file 3** Figure S3 Click here for file
