# Supplementary material for: Functional repertoire, molecular pathways and diseases associated with 3D domain swapping in the human proteome
Source: J Clin Bioinforma. 2012 Apr 3;2:8. doi: 10.1186/2043-9113-2-8 (PMC3508620; doi:10.1186/2043-9113-2-8)
Supplement: Additional file 1 — Supplementary Table 1 [file 2043-9113-2-8-S1.PDF]

**Accession**	**Entry name**	**Protein names**	**Gene synonyms**
P01008	ANT3_HUMAN	Antithrombin-III (ATIII) (Serpin C1)	*SERPINC1, AT3, PRO0309*
Q07817	B2CL1_HUMAN	Bcl-2-like protein 1 (Bcl2-L-1) (Apoptosis regulator Bcl-X)	*BCL2L1, BCL2L, BCLX*
Q14457	BECN1_HUMAN	Beclin-1 (Coiled-coil myosin-like BCL2-interacting protein) (Protein GT197)	*BECN1, GT197*
Q96EP1	CHFR_HUMAN	E3 ubiquitin-protein ligase CHFR (EC 6.3.2.-) (Checkpoint with forkhead and RING finger domains protein) (RING finger protein 196)	*CHFR, RNF196*
P33552	CKS2_HUMAN	Cyclin-dependent kinases regulatory subunit 2 (CKS-2)	*CKS2*
P01040	CYTA_HUMAN	Cystatin-A (Cystatin-AS) (Stefin-A)	*CSTA, STF1, STFA*
P04080	CYTB_HUMAN	Cystatin-B (CPI-B) (Liver thiol proteinase inhibitor) (Stefin-B)	*CSTB, CST6, STFB*
P01034	CYTC_HUMAN	Cystatin-C (Cystatin-3) (Gamma-trace) (Neuroendocrine basic polypeptide) (Post-gamma-globulin)	*CST3*
Q96C86	DCPS_HUMAN	Scavenger mRNA-decapping enzyme DcpS (EC 3.-.-.-) (DCS-1) (Hint-related 7meGMP-directed hydrolase) (Histidine triad protein member 5) (HINT-5)	*DCPS, DCS1, HINT5, HSPC015*
Q14203	DCTN1_HUMAN	Dynactin subunit 1 (150 kDa dynein-associated polypeptide) (DAP-150) (DP-150) (p135) (p150-glued)	*DCTN1*
P11532	DMD_HUMAN	Dystrophin	*DMD*
Q05397	FAK1_H	Focal adhesion kinase 1 (FADK 1) (EC 2.7.10.2)	*PTK2, FAK,*

	UMAN	(Protein-tyrosine kinase 2) (pp125FAK)	*FAK1*
O15409	FOXP2_HUMAN	Forkhead box protein P2 (CAG repeat protein 44) (Trinucleotide repeat-containing gene 10 protein)	*FOXP2, CAGH44, TNRC10*
P04150	GCR_HUMAN	Glucocorticoid receptor (GR) (Nuclear receptor subfamily 3 group C member 1)	*NR3C1, GRL*
P62993	GRB2_HUMAN	Growth factor receptor-bound protein 2 (Adapter protein GRB2) (Protein Ash) (SH2/SH3 adapter GRB2)	*GRB2, ASH*
P51858	HDGF_HUMAN	Hepatoma-derived growth factor (HDGF) (High mobility group protein 1-like 2) (HMG-1L2)	*HDGF, HMG1L2*
P14210	HGF_HUMAN	Hepatocyte growth factor (Hepatopoeitin-A) (Scatter factor) (SF) [Cleaved into: Hepatocyte growth factor alpha chain; Hepatocyte growth factor beta chain]	*HGF, HPTA*
P34913	HYES_HUMAN	Epoxide hydrolase 2 (EC 3.3.2.10) (Cytosolic epoxide hydrolase) (CEH) (Epoxide hydratase) (Soluble epoxide hydrolase) (SEH)	*EPHX2*
P22301	IL10_HUMAN	Interleukin-10 (IL-10) (Cytokine synthesis inhibitory factor) (CSIF)	*IL10*
P05113	IL5_HUMAN	Interleukin-5 (IL-5) (B-cell differentiation factor I) (Eosinophil differentiation factor) (T-cell replacing factor) (TRF)	*IL5*
Q04760	LGUL_HUMAN	Lactoylglutathione lyase (EC 4.4.1.5) (Aldoketomutase) (Glyoxalase I) (Glx I) (Ketone-aldehyde mutase) (Methylglyoxalase) (S-D-lactoylglutathione methylglyoxal lyase)	*GLO1*
Q5S007	LRRK2_HUMAN	Leucine-rich repeat serine/threonine-protein kinase 2 (EC 2.7.11.1) (Dardarin)	*LRRK2, PARK8*
P08581	MET_HU	Hepatocyte growth factor receptor (HGF	*MET*

	MAN	receptor) (EC 2.7.10.1) (HGF/SF receptor) (Proto-oncogene c-Met) (Scatter factor receptor) (SF receptor) (Tyrosine-protein kinase Met)	
P22897	MRC1_HUMAN	Macrophage mannose receptor 1 (MMR) (C-type lectin domain family 13 member D) (CD antigen CD206)	*MRC1, CLEC13D*
P28698	MZF1_HUMAN	Myeloid zinc finger 1 (MZF-1) (Zinc finger and SCAN domain-containing protein 6) (Zinc finger protein 42)	*MZF1, MZF, ZNF42, ZSCAN6*
O15394	NCAM2_HUMAN	Neural cell adhesion molecule 2 (N-CAM-2) (NCAM-2)	*NCAM2, NCAM21*
Q15596	NCOA2_HUMAN	Nuclear receptor coactivator 2 (NCoA-2) (Class E basic helix-loop-helix protein 75) (bHLHe75) (Transcriptional intermediary factor 2) (hTIF2)	*NCOA2, BHLHE75, TIF2*
Q9Y239	NOD1_HUMAN	Nucleotide-binding oligomerization domain-containing protein 1 (Caspase recruitment domain-containing protein 4)	*NOD1, CARD4*
P04629	NTRK1_HUMAN	High affinity nerve growth factor receptor (EC 2.7.10.1) (Neurotrophic tyrosine kinase receptor type 1) (TRK1-transforming tyrosine kinase protein) (p140-TrkA) (Trk-A)	*NTRK1, TRK*
Q16620	NTRK2_HUMAN	BDNF/NT-3 growth factors receptor (EC 2.7.10.1) (GP145-TrkB) (Trk-B) (Neurotrophic tyrosine kinase receptor type 2) (TrkB tyrosine kinase)	*NTRK2, TRKB*
Q16288	NTRK3_HUMAN	NT-3 growth factor receptor (EC 2.7.10.1) (GP145-TrkC) (Trk-C) (Neurotrophic tyrosine kinase receptor type 3) (TrkC tyrosine kinase)	*NTRK3, TRKC*
Q9UKK9	NUDT5_HUMAN	ADP-sugar pyrophosphatase (EC 3.6.1.-) (EC 3.6.1.13) (Nucleoside diphosphate-linked moiety X motif 5) (Nudix motif 5) (YSA1H)	*NUDT5, HSPC115*

P10515	ODP2_HUMAN	Dihydrolipoyllysine-residue acetyltransferase component of pyruvate dehydrogenase complex, mitochondrial (EC 2.3.1.12) (70 kDa mitochondrial autoantigen of primary biliary cirrhosis) (PBC) (Dihydrolipoamide acetyltransferase component of pyruvate dehydrogenase complex) (M2 antigen complex 70 kDa subunit) (Pyruvate dehydrogenase complex component E2) (PDC-E2) (PDCE2)	*DLAT, DLTA*
Q15120	PDK3_HUMAN	[Pyruvate dehydrogenase [lipoamide]] kinase isozyme 3, mitochondrial (EC 2.7.11.2) (Pyruvate dehydrogenase kinase isoform 3)	*PDK3*
P00439	PH4H_HUMAN	Phenylalanine-4-hydroxylase (PAH) (EC 1.14.16.1) (Phe-4-monooxygenase)	*PAH*
P04156	PRIO_HUMAN	Major prion protein (PrP) (ASCR) (PrP27-30) (PrP33-35C) (CD antigen CD230)	*PRNP, PRIP, PRP*
P49758	RGS6_HUMAN	Regulator of G-protein signaling 6 (RGS6) (S914)	*RGS6*
P07998	RNAS1_HUMAN	Ribonuclease pancreatic (EC 3.1.27.5) (HP-RNase) (RIB-1) (RNase UpI-1) (Ribonuclease 1) (RNase 1) (Ribonuclease A) (RNase A)	*RNASE1, RIB1, RNS1*
P07602	SAP_HUMAN	Proactivator polypeptide [Cleaved into: Saposin-A (Protein A); Saposin-B-Val; Saposin-B (Cerebroside sulfate activator) (CSAct) (Dispersin) (Sphingolipid activator protein 1) (SAP-1) (Sulfatide/GM1 activator); Saposin-C (A1 activator) (Co-beta-glucosidase) (Glucosylceramidase activator) (Sphingolipid activator protein 2) (SAP-2); Saposin-D (Component C) (Protein C)]	*PSAP, GLBA, SAP1*
Q9NP99	TREM1_	Triggering receptor expressed on myeloid cells 1	*TREM1*

	HUMAN	(TREM-1) (Triggering receptor expressed on monocytes 1) (CD antigen CD354)	
P40818	UBP8_HUMAN	Ubiquitin carboxyl-terminal hydrolase 8 (EC 3.4.19.12) (Deubiquitinating enzyme 8) (Ubiquitin isopeptidase Y) (hUBPy) (Ubiquitin thiolesterase 8) (Ubiquitin-specific-processing protease 8)	*USP8, KIAA0055, UBPY*
Q05516	ZBT16_HUMAN	Zinc finger and BTB domain-containing protein 16 (Promyelocytic leukemia zinc finger protein) (Zinc finger protein 145) (Zinc finger protein PLZF)	*ZBTB16, PLZF, ZNF145*
Q15697	ZN174_HUMAN	Zinc finger protein 174 (AW-1) (Zinc finger and SCAN domain-containing protein 8)	*ZNF174, ZSCAN8*
Q07157	ZO1_HUMAN	Tight junction protein ZO-1 (Tight junction protein 1) (Zona occludens protein 1) (Zonula occludens protein 1)	*TJP1, ZO1*
Q9UDY2	ZO2_HUMAN	Tight junction protein ZO-2 (Tight junction protein 2) (Zona occludens protein 2) (Zonula occludens protein 2)	*TJP2, X104, ZO2*

Supplementary Table 1: List of human proteins in the dataset used for genome-wide enrichment analysis using Gene Ontology annotations of human gene products
