# Supplementary figures and images for: Functional repertoire, molecular pathways and diseases associated with 3D domain swapping in the human proteome
Source: J Clin Bioinforma. 2012 Apr 3;2:8. doi: 10.1186/2043-9113-2-8 (PMC3508620; doi:10.1186/2043-9113-2-8)

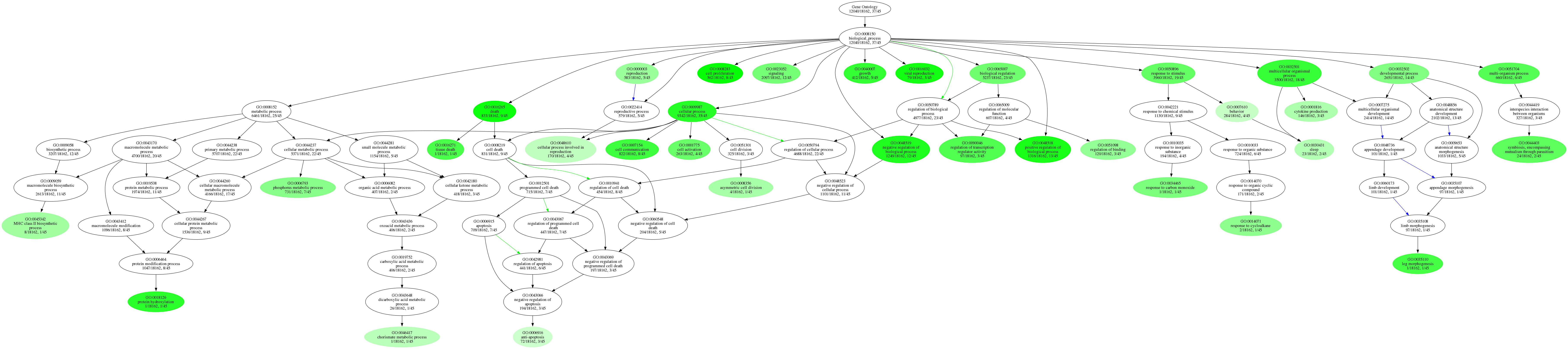

Supplement: Additional file 2 — Figure S2 [file 2043-9113-2-8-S2.PNG]

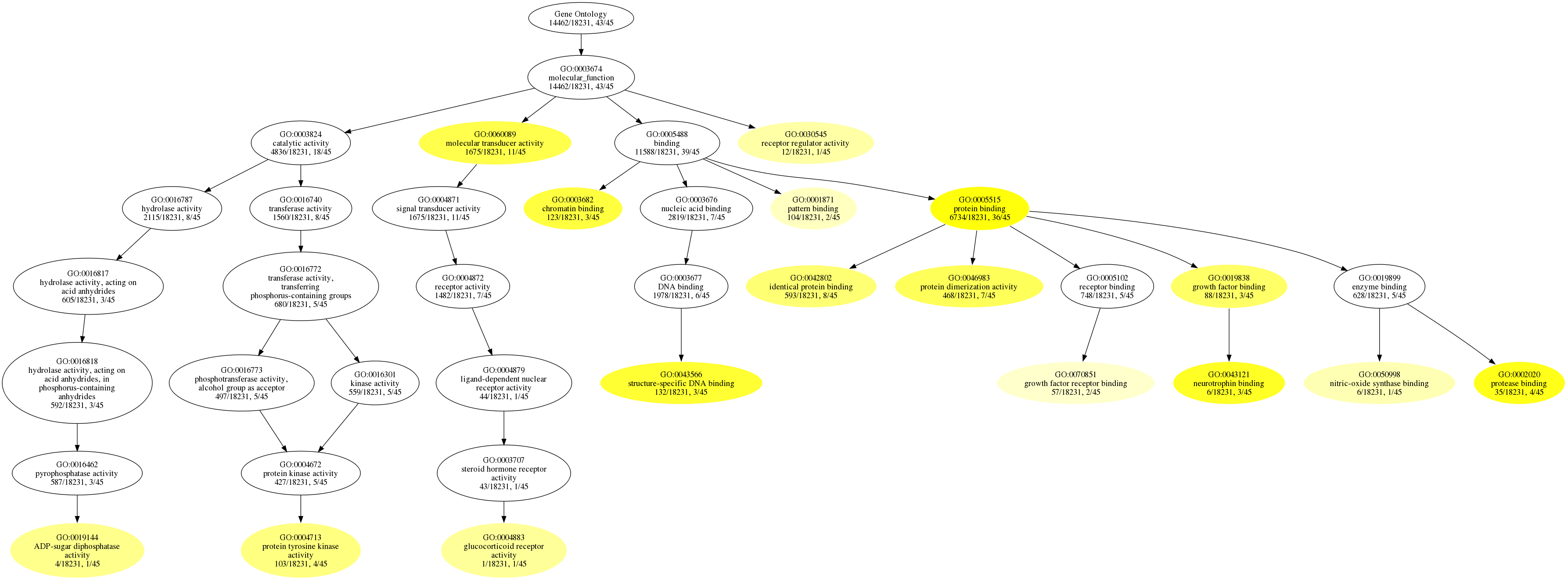

Supplement: Additional file 3 — Figure S3 [file 2043-9113-2-8-S3.PNG]
